# Comparison of atrial fibrillation predictors in patients with acute coronary syndrome using ticagrelor or clopidogrel

**DOI:** 10.3906/sag-1903-188

**Published:** 2019-10-24

**Authors:** Engin ALGÜL, Hamza SUNMAN, Muhammet DURAL, İlkin GULİYEV, Mert AKER, Mehmet Ali FELEKOĞLU, Mehmet ERAT, Murat TULMAÇ, Sadık AÇIKEL, Tolga ÇİMEN

**Affiliations:** 1 Cardiology Clinics, Bitlis State Hospital, Bitlis Turkey; 2 Department of Cardiology, University of Health Sciences, Dışkapı Yıldırım Beyazıt Training and Research Hospital, Ankara Turkey; 3 Department of Cardiology, Eskişehir Osmangazi University, Eskişehir Turkey; 4 Cardiology Clinics, Dr. Ersin Arslan Training and Research Hospital, Gaziantep Turkey

**Keywords:** Acute coronary syndrome, atrial fibrillation, ticagrelor

## Abstract

**Background/aim:**

Ticagrelor is a drug widely used in patients with acute coronary syndromes (ACS) that specifically increases the plasma level of adenosine, which is likely to cause atrial fibrillation (AF). Therefore, in this study we aimed to investigate the electrocardiographic and echocardiographic predictors of AF development after P2Y12 receptor antagonists in ACS patients.

**Materials and methods:**

This cross-sectional study included 831 patients with ACS (486 [58.5%] with ST elevated myocardial infarction [STEMI] and 345 [41.5%] with non-ST elevated myocardial infarction [NSTEMI]). Patients were divided into ticagrelor (n = 410) and clopidogrel (n = 421) groups. P wave properties including P wave dispersion and atrial electromechanical conduction properties were measured as AF predictors with surface ECG and tissue Doppler imaging.

**Results:**

Baseline characteristics such as age, sex, heart rate, blood pressure, and laboratory parameters were almost the same in the ticagrelor and clopidogrel groups. The statistical analysis showed no significant difference in P wave dispersion (PWD) between ticagrelor and clopidogrel groups (40.98 ± 12 ms versus 40.06 ± 12 ms, P = 0.304). Subgroups analysis according to ACS types also showed no significant difference in PWD (NSTEMI: 41.16 ± 13.8 ms versus 40.76 ± 13.55 ms, P = 0.799; STEMI: 40.9 ± 12.62 ms versus 39.19 ± 11.18 ms, P = 0.132). In addition, we did not find significant difference in atrial electromechanical delay (EMD) with tissue Doppler imaging (interatrial EMD 24.11 ± 3.06 ms versus 24.46 ± 3.23 ms, P = 0.279).

**Conclusion:**

In conclusion, we did not find any difference in detailed electrocardiographic and echocardiographic parameters as AF predictors between ticagrelor and clopidogrel groups in patients with ACS.

## 1. Introduction

Acute coronary syndromes (ACS) are one of the major causes of mortality and morbidity worldwide. Current guidelines recommend dual antiplatelet therapy in patients with ACS [1,2]. Ticagrelor, one of the relatively new drugs used in ACS, is a reversible and direct-acting oral antagonist of adenosine diphosphate receptor P2Y12, and it was found superior over clopidogrel in the PLATO trial [3]. Although the benefit of ticagrelor has been attributed mostly to its faster, greater, and more consistent P2Y12 inhibition compared to clopidogrel, continuity of growing benefits of ticagrelor and its effect on reduction of cardiovascular mortality in the PLATO trial make it different from other P2Y12-ADP receptor blockers [3–5]. These differences led to the hypothesis that ticagrelor has pleiotropic properties and nonplatelet directed mechanisms of action. These effects of ticagrelor have been mostly attributed to increased half-life and plasma concentration of adenosine [6,7].

Adenosine is a purine nucleoside primarily produced by endothelial cells [8] and it has a number of effects, such as coronary vasodilatation [9], inhibition of platelet aggregation [10], modulation of inflammation [11], reduced ischemia/reperfusion injury [12,13], and reduced atrioventricular conduction [14]. Besides some positive effects, it is also known that adenosine has the potential to cause atrial fibrillation (AF) [15–17]. In addition, there is a case report in the literature suggesting that ticagrelor could cause AF, a possible mechanism of which is increased plasma adenosine level [18]. However, there are no studies in the literature investigating the risk of AF in patients treated with ticagrelor. In this study, we aimed to determine whether ticagrelor predisposes to AF in ACS patients by using surrogate electro and echocardiographic parameters.

## 2. Materials and methods

This cross-sectional study was conducted between January 2016 and February 2017 on patients diagnosed with ACS, which consists of ST elevated myocardial infarction (STEMI) and non-ST elevated myocardial infarction (NSTEMI). STEMI is defined as having a typical angina that lasts 20 min or longer and with STEMI criteria in ECG [2]. Non-ST-elevation myocardial infarction is defined as a rise in troponin level (troponin-I > 0.06 ng/mL) with typical chest pain without STEMI criteria in ECG [1]. The treatment of the patients was arranged in line with the European Society of Cardiology guidelines. Patients were given 180 mg ticagrelor as the loading dose in the ticagrelor group. Angiotensin converting enzyme inhibitors, beta blockers, and statins were started in all patients without contraindication within the first 24 h after diagnosis. Patients were treated with percutaneous coronary intervention (stent implantation or balloon angioplasty). Patients who needed coronary bypass surgery were not included in the study. The other exclusion criteria were as follows: atrial infarction diagnostic criteria described by Liu et al. [19], a history of AF, use of antiarrhythmic drug other than beta-blockers, renal dysfunction (creatinine >1.5 mg/dL), severe valvular heart disease, permanent pacemaker, cerebrovascular disease, and the need for mechanical ventilation. The study was conducted following the principles of the Declaration of Helsinki for Human Research and approved by the institutional ethics committee.

Electro-echocardiographic evaluation was performed on patients who had been treated for a median of 2.5 days. A 12-lead surface ECG was obtained from all study participants nearly 2 h after the last dose of the clopidogrel/ticagrelor in the supine position before discharge and analyses were done with this ECG [20]. The point at which the first atrial deflection crossed the isoelectric line was defined as the beginning of the P wave and the return to baseline was considered as the end of the P wave.  Pmax and Pmin durations were measured for all patients in all 12 leads on the ECG. The difference between Pmax and Pmin durations on the ECG was defined as P wave dispersion (PWD) [21]. The laboratory tests included complete blood count, fasting glucose level, lipid profile, troponin level, liver, kidney, and thyroid function tests. The weight and height of the participants were measured, and the body mass index (BMI) (kg/m2) was calculated using the following formula: BMI = weight/(height)2. Echocardiographic examination and evaluation of patients were performed using Philips Healthcare iE33 xMATRIX Echocardiography (Philips Medical System, Andover, MA, USA) with an S5–1 transducer before discharge. Examinations were performed by a single experienced cardiologist who was blinded to the patients and their characteristics. Evaluation of the patients was performed in the left lateral decubitus position. A continuous one-lead ECG was obtained during all examinations. The average of three consecutive beats was used to calculate the associated parameters. M-mode echocardiography was used in the parasternal long-axis view to measure basic echocardiographic parameters, such as the left atrium (LA), left ventricular (LV) end-systolic and end-diastolic dimensions, LV ejection fraction (EF), and diastolic LV septal and posterior wall thickness. 

Electromechanical properties of the atria were determined by tissue Doppler imaging. Before the study, the Nyquist limit was adjusted to 15–20 cm/s and the monitor sweep speed was set at 50–100 mm/s to optimize the spectral display myocardial velocities. The pulsed doppler sample volume was placed at the LV lateral and septal mitral annulus, and subsequently at the septal mitral annulus and right ventricular tricuspid annulus in apical four chamber view. Atrial electromechanical delay (EMD) was considered as the time interval from the onset of P wave on ECG to the beginning of “A”-wave in tissue Doppler (PA). PA interval, which was measured from the lateral mitral, septal mitral, and tricuspid annulus was called PA lateral, PA septum, and PA tricuspid, respectively. PA intervals between the lateral and right ventricular annulus were accepted as interatrial EMD, the difference between the septal and tricuspid PA intervals as right intraatrial EMD, and the difference between the lateral and septal PA intervals as left intraatrial EMD. 

The data were analyzed using SPSS 18.0 statistics package (SPSS Inc., Chicago, IL, USA). Normally distributed continuous variables were expressed as means ± standard deviation, while continuous variables with a nonnormal distribution were expressed as median and interquartile ranges (IQR). Student’s t-test or the Mann–Whitney U test were used to compare the means or medians of groups, respectively. Categorical data were expressed as proportions and compared using the chi-square test. The relationship between antiplatelet types and P wave duration, PWD, PR interval, P wave axis, and EMD values was assessed by ANCOVA analysis by removing the effects of confounding factors that were significant in the antiplatelet and ACS types. P-values less than 0.05 were considered statistically significant.

## 3. Results

A total of 1036 patients were assessed with a diagnosis of ACS. Eighty-eight patients were excluded because of the atrial infarction criteria and 45 patients due to the history of AF. After exclusion of the patients with renal dysfunction (creatinine >1.5 mg/dL), severe valvular heart disease, permanent pacemaker, cerebrovascular disease, and the need for mechanical ventilation, 831 patients remained for further analysis. STEMI was found in 486 (58.5%) and NSTEMI in 345 (41.5%) patients. All patients were divided into two groups according to receiving ticagrelor (410, 49.3%) or clopidogrel (421, 50.6%). The rate of patients diagnosed with STEMI in the ticagrelor group was found to be higher than in the clopidogrel group (72.2% versus 45.2%, P < 0.001). Table 1 summarizes the patients’ baseline characteristics. The groups had no significant differences in terms of age, sex, BMI, heart rate, blood pressure, creatinine, alanine amino transferase, hemoglobin, thyroid-stimulating hormone levels, LA diameter, and LVEF. As shown in Table 1, LDL, HDL, and 24th hour troponin levels were significantly higher in the ticagrelor group. 

In the analysis of total population data, there was no statistically significant difference in PWD between ticagrelor and clopidogrel groups (40.98 ± 12 ms versus 40.06 ± 12 ms, P = 0.304) (Table 2). The other electrocardiographic predictors of AF, such as PR interval and P wave axis, also showed no significant difference (P = 0.553 and P = 0.168, respectively). Subgroups analysis according to ACS types also showed no significant difference in PWD (NSTEMI: 41.16 ± 13.8 ms versus 40.76 ± 13.55 ms, P = 0.799; STEMI: 40.9 ± 12.62 ms versus 39.19 ± 11.18 ms, P = 0.132). Echocardiographic parameters are shown in Table 2. Like electrocardiographic parameters, we did not find any differences in echocardiographic predictors. There was no statistically significant difference in left intraatrial EMD, right intraatrial EMD, and interatrial EMD between the groups (12.3 ± 2.73 ms versus 12.3 ± 2.73 ms, P = 0.314; 11.81 ± 1.39 ms versus 11.86 ± 1.33 ms, P = 0.693; 24.11 ± 3.06 ms versus 24.46 ± 3.23 ms, P = 0.279). In addition, there was no statistically significant difference in echocardiographic predictors of AF in the subgroups according to ACS types. The mean interatrial EMD values of ticagrelor and clopidogrel groups were found as 24.53 ± 3.74 ms and 24.86 ± 3.33 ms in NSTEMI (P = 0.644) and as 23.98 ± 2.82 ms versus 24.23 ± 3.16 ms (P = 0.493) in STEMI subgroup (P = 0.493).

**Table 1:** Baseline characteristics of the patients.

	Total n = 831	Ticagrelor n = 410	Clopidogrel n = 421	P
Age (years), mean ± SD	61.79 ± 11.97	61.04 ± 11.26	62.51 ± 12.59	0.076
Female, n (%)	223 (26.8)	103 (25.1)	120 (28.5)	0.271
Diabetes mellitus, n (%)	247 (29.7)	114 (27.8)	133 (31.6)	0.232
Hypertension, n (%)	420 (50.5)	199 (48.5)	221 (52.1)	0.254
Smoking, n (%)	420 (50.5)	220 (53.7)	200 (47.5)	0.076
Previous history of CAD, n (%)	273 (32.9)	123 (30.0)	150 (35.6)	0.084
ACS type, n (%)				
NSTEMI	345 (41.5)	114 (27.8)	231 (54.9)	<0.001
STEMI	486 (58.5)	296 (72.2)	190 (45.1)	
BMI (kg/m²), mean ± SD	27.75 ± 4.56	27.79 ± 4.69	27.70 ± 4.44	0.764
SBP (mmHg), mean ± SD	134.65 ± 26.17	136.3 ± 26.62	133.06 ± 25.67	0.077
DBP (mmHg), mean ± SD	79.74 ± 13.83	80.00 ± 14.15	79.48 ± 13.52	0.592
Heart rate (bpm), mean ± SD	75.6 ± 13.68	74.91 ± 13.8	76.26 ± 13.55	0.159
Glucose (mg/dL), median (IQR)	122 (100–165)	126 (102–168)	118 (99–162.75)	0.084
Hemoglobin (g/dL), mean ± SD	14.41 ± 1.80	14.5 ± 1.74	14.31 ± 1.85	0.139
Platelet (103/µL), mean ± SD	251.57 ± 75.59	253.89 ± 78.38	249.3 ± 72.78	0.382
Creatinine (mg/dL), mean ± SD	1.05 ± 0.22	1.05 ± 0.20	1.05 ± 0.24	0.857
Cholesterol (mg/dL), mean ± SD	191.22 ± 45.15	193.21 ± 44.15	189.21 ± 46.11	0.222
LDL (mg/dL), mean ± SD	128.85 ± 35.15	131.33 ± 34.39	126.37 ± 35.75	0.045
HDL (mg/dL), mean ± SD	39.45 ± 8.68	40.15 ± 8.54	38.75 ± 8.77	0.024
Triglyceride (mg/dL), median (IQR)	136 (98–195)	138 (99.25–195.75)	133 (97.5–194)	0.741
TSH (mIU/L), median (IQR)	1.23 (0.71–1.87)	1.22 (0.72–1.83)	1.24 (0.70–1.92)	0.914
Potassium (mmol/L), mean ± SD	4.1 ± 0.44	4.10 ± 0.43	4.11 ± 0.45	0.800
Calcium (mg/dL), mean ± SD	9.11 ± 0.6	9.15 ± 0.58	9.08 ± 0.61	0.083
Albumin (g/dL), mean ± SD	3.84 ± 0.5	3.87 ± 0.39	3.81 ± 0.58	0.158
Troponin (ng/mL), median (IQR)	11.59 (1.84–40.59)	14.15 (3.28–54.95)	9.61 (1.27–35.5)	0.003
LVEF (%), mean ± SD	48.8 ± 9.65	48.33 ± 8.98	49.27 ± 10.27	0.168
LVEDD (cm), mean ± SD	5.44 ± 0.37	5.44 ± 0.32	5.44 ± 0.40	0.808
Left atrium (cm), mean ± SD	3.68 ± 0.42	3.65 ± 0.38	3.70 ± 0.46	0.167

The data without normal distribution is presented as median (interquartile range).ACS: acute coronary syndrome; BMI: body mass index; CAD: coronary artery disease; DBP: diastolic blood pressure; HDL: high density lipoprotein; IQR: interquartile range; LDL: low density lipoprotein; LVEDD: left ventricular end-diastolic diameter; LVEF: left ventricular ejection fraction; NSTEMI: non-ST elevated myocardial infarction; SBP: systolic blood pressure; SD: standard deviation; STEMI: ST elevated myocardial infarction; TSH: thyrotropin stimulating hormone; WBC: white blood cell

**Table 2:** Comparison of electrocardiographic and echocardiographic data by type of acute coronary syndrome.

	NSTEMI			STEMI			Total			Ticagrelorn = 114	Clopidogreln = 231	P	Ticagrelorn = 296	Clopidogreln = 190	P	Ticagrelorn = 410	Clopidogreln = 421	P
	Pmax (ms), mean ± SD	108.62 ± 14.48	108.22 ± 16.67	0.820	106.62 ± 16.26	105.86 ± 16.2	0.617	107.17 ± 15.79	107.16 ± 16.48	0.993
Pmin (ms), mean ± SD	67.46 ± 13.95	67.46 ± 15.79	0.999	65.72 ± 13.54	66.67 ± 15.3	0.477	66.2 ± 13.66	67.1 ± 15.56	0.376
PWD (ms), mean ± SD	41.16 ± 13.8	40.76 ± 13.55	0.799	40.9 ± 12.62	39.19 ± 11.18	0.132	40.98 ± 12.94	40.06 ± 12.56	0.304
PR interval (ms), mean ± SD	156.75 ± 21.16	164.72 ± 26.70	0.135	163.70 ± 23.41	162.57 ± 29.11	0.814	162.10 ± 23.03	164.00 ± 27.40	0.553
P wave axis (degree), median (IQR)	46.0 (35.0–61.5)	45.0 (29.0–61.3)	0.231	49.0 (36.8–63.0)	49.0 (29.0–71.0)	0.673	48.0 (37.0–63.0)	47.0 (29.0–63.5)	0.168
QT interval (ms), mean ± SD	388.3 ± 41.3	389.38 ± 36.81	0.829	391.36 ± 37.54	384.52 ± 33.94	0.063	390.54 ± 38.55	387.06 ± 35.5	0.226
QTc interval (ms), mean ± SD	418.34 ± 32.14	421.69 ± 32.3	0.428	425.17 ± 32.53	422.73 ± 31.3	0.453	423.33 ± 32.52	422.19 ± 31.78	0.645
QRS duration (ms), mean ± SD	98.6 ± 12.26	101.93 ± 18.91	0.086	98.9 ± 14.73	98.34 ± 13.69	0.289	99.54 ± 14.1	100.22 ± 16.7	0.571
Ejection fraction (%), mean ± SD	53.65 ± 8.66	52.47 ± 9.88	0.283	46.22 ± 8.22	45.33 ± 9.35	0.281	48.33 ± 8.98	49.27 ± 10.27	0.168
Left atrium (mm), mean ± SD	3.70 ± 0.41	3.79 ± 0.46	0.066	3.64 ± 0.37	3.58 ± 0.43	0.099	3.65 ± 0.38	3.70 ± 0.46	0.167
Posterior wall (cm), mean ± SD	1.00 ± 0.13	1.03 ± 0.13	0.123	1.03 ± 0.11	1.01 ± 0.12	0.250	1.02 ± 0.12	1.02 ± 0.13	0.897
Septal wall (cm), mean ± SD	1.06 ± 0.16	1.10 ± 0.15	0.044	1.09 ± 0.13	1.06 ± 0.14	0.006	1.09 ± 0.14	1.08 ± 0.15	0.721
PA lateral (ms), mean ± SD	49.02 ± 6.12	48.51 ± 4.36	0.635	47.03 ± 5.35	46.74 ± 3.98	0.611	47.49 ± 5.58	47.38 ± 4.20	0.820
PA septal (ms), mean ± SD	36.37 ± 5.74	35.43 ± 3.59	0.337	34.84 ± 4.60	34.42 ± 2.82	0.368	35.19 ± 4.91	34.78 ± 3.15	0.337
PA tricuspid (ms), mean ± SD	24.49 ± 5.80	23.66 ± 3.36	0.396	23.06 ± 4.47	22.51 ± 2.51	0.213	23.39 ± 4.83	22.92 ± 2.89	0.259
Interatrial EMD (ms), mean ± SD	24.53 ± 3.74	24.86 ± 3.33	0.644	23.98 ± 2.82	24.23 ± 3.16	0.493	24.11 ± 3.06	24.46 ± 3.23	0.279
Right intraatrial EMD (ms), mean ± SD	11.88 ± 1.56	11.77 ± 1.23	0.672	11.78 ± 1.34	11.91 ± 1.39	0.440	11.81 ± 1.39	11.86 ± 1.33	0.693
Left intraatrial EMD (ms), mean ± SD	12.65 ± 3.52	13.09 ± 3.24	0.504	12.20 ± 2.45	12.32 ± 2.77	0.697	12.3 ± 2.73	12.59 ± 2.96	0.314

The data without normal distribution is presented as median (interquartile range).EMD: electromechanical delay; NSTEMI: non-ST elevated myocardial infarction; Pmax: the longest P wave duration; Pmin: the shortest P wave duration; PWD: P wave dispersion; SD: standard deviation; STEMI: ST elevated myocardial infarction

The relationship between antiplatelet types and P wave duration, PWD and EMD values, was evaluated by ANCOVA analysis by removing the effects of confounding factors (age, body mass index, hypertension, CAD history, troponin, QTc, left atrial diameter, deceleration time, and left ventricular iso-volumetric relaxation time) that were found to be significant between the antiplatelet groups and the ACS subgroups. When the effects of confounding factors were removed, there was no significant relationship between antiplatelet use and AF predictors (P > 0.05).

From the date of treatment initiation, subgroup analysis was performed in 66 (16.09%) patients with side effects (mainly dyspnea, rarely others) related to ticagrelor. There was no significant difference in terms of AF predictors in patients with side effects related to ticagrelor when compared to the group receiving clopidogrel or ticagrelor without side effects. PWD was 38.79 ± 12.28 ms in patients receiving ticagrelor that had side effects, while this value was 41.44 ± 13.17 ms in patients without side effects (P = 0.279) and 40.34 ± 13.14 ms in clopidogrel group (P = 0.657). Interatrial EMD was 24.23 ± 2.79 ms in patients receiving ticagrelor and had side effects, while this value was 24.18 ± 3.21 ms in patients without side effects (P = 0.998) and 24.35 ± 3.01 ms in clopidogrel group (P = 0.990).

## 4. Discussion

Our study was designed to find out whether ticagrelor could change AF predictors. We did not find a statistically significant difference in PWD, interatrial and intraatrial EMD durations between ticagrelor and clopidogrel groups in patients with ACS.

In ACS patients, AF is seen at a high rate (approximately 10%) compared to the normal population, and this increase has been associated with long-term increased morbidity and mortality [22,23]. AF development in patients with ACS is mostly attributed to ischemia and decreased atrial perfusion, increased left ventricular end-diastolic pressure, increased left atrial pressure, diastolic dysfunction, and autonomic regulatory disturbances [24]. In addition, recently, inflammation and neurohumoral factors have been shown to be associated with AF development in patients with ACS [24]. In our study, there were no significant differences between the groups in parameters that could affect the development of AF such as LA diameter, systolic and diastolic functions of LV. Furthermore, patients with atrial infarction criteria on ECG were excluded from the study to reduce the possible confounding effects of atrial ischemia [19]. Apart from the normal course of ACS, there is a case report indicating that ticagrelor, which increases plasma concentration of adenosine, may cause AF [18]. PLATO trial and some case reports have shown that ticagrelor causes bradyarrhythmias, such as atrioventricular block and sinus node pause [3,25–27]. The most probable mechanism for these bradycardic events appears to be increasing plasma concentration of adenosine. Besides the bradycardic effects of adenosine, it is known that intravenous adenosine administration could cause spontaneous AF [15]. Moreover, endogenous production of adenosine during metabolic stress conditions has been suggested as a trigger of AF [28,29]. Although the mechanism is not clearly understood, this phenomenon is thought to be mediated by adenosine’s effects on shortening atrial action potential duration and refractoriness [30]. Because adenosine has little effect on atrial conduction velocity, the net effect of adenosine, therefore, is to shorten the wavelength of activation, thereby potentiating AF. This cellular electrophysiological effect is mediated by its specific G protein-coupled adenosine A1 receptor and this ligand activates the heterotrimeric protein Gi/o, which then activates the inward rectifying K+ current, *I*KAdo. In addition to that, adenosine has other effects that may promote arrhythmogenesis. Adenosine has sympathoexcitatory effects mediated through baroreflex activation and chemoreceptor stimulation [30]. Adenosine can also hyperpolarize dormant pulmonary vein myocytes and increase excitability, as well as trigger pulmonary vein ectopy [31,32]. It has been shown that ticagrelor increases plasma adenosine levels as early as six hours after the loading dose in patients with ACS [33]. Therefore, the main point of our study was to determine whether additional increase in adenosine, which is caused by ticagrelor, would increase the susceptibility to AF. 

It has been shown that PWD and Pmax are predictors of AF [34]. Normal value for PWD is defined as 29 ± 9 ms in the literature, while a PWD > 40 ms leads to atrial tachyarrhythmias [34,35]. Rosiak et al. also demonstrated that PWD is predictive of AF in STEMI patients and that a value of >25 ms is associated with increased risk for AF [36]. In our study, PWD was 40.98 ± 12 in the ticagrelor group and 40.06 ± 12 in the clopidogrel group, and no statistical difference was found between the two groups. PWD value determined in our study is higher than the PWD values in non-ACS patients in the literature, but the patient groups show some differences. In particular, the high value of PWD may explain the fact that since our patient group included ACS patients, additional comorbidities were common and LVEF was lower than other studies in the literature. Thus, Ding et al. found that patients with cardiac resynchronization therapy had high PWD values and showed a decrease in PWD when EF was improved [37]. Besides electrocardiographic parameters, we also looked at echocardiographic parameters, which were shown to predict AF [38,39]. Likewise, in electrocardiographic analysis, there was no statistical difference between the interatrial, right and left intraatrial electro-mechanic delays that were measured by TDI between ticagrelor and clopidogrel groups. 

 Although recent investigations have shown that dyspnea is caused by direct P2Y12-inhibitory effect on the central nervous system, in our study patients with side effects due to ticagrelor were also considered as subgroups due to probably increased adenosine levels than those without side effects [40]. However, subgroup analyses for AF predictors showed no significant difference in patients with or without side effects [20]. 

To the best of our knowledge, this is the first study investigating the association between ticagrelor and AF predictors. Nevertheless, the lack of follow-up in terms of future arrhythmic episodes is among the major limitations of our study. Reliable methods for detecting AF could be used, such as long-term rhythm monitoring. In addition, the lack of difference between the ticagrelor and clopidogrel groups in terms of AF predictors may be attributed to several causes. For example, possible atrial structural disturbances due to ACS and having multiple risk factors may have masked potential effects of adenosine on AF development. There were also some differences in frequency of ACS types and troponin levels in the two study groups. Furthermore, the increase in adenosine level caused by ticagrelor might not be sufficient to alter the AF predictors we have investigated. It has also been shown in the literature that adenosine causes AF in a dose-dependent AF [14].

In conclusion, in this study, we found that there was no significant difference in electro-echocardiographic AF predictors such as PWD and EMD in ACS patients who received ticagrelor or clopidogrel. In addition, there was no difference even in patients with side effects, suggesting that the possible increase in adenosine level did not lead to AF susceptibility.
